# Biomarkers and overall survival in patients with advanced hepatocellular carcinoma treated with TGF-βRI inhibitor galunisertib

**DOI:** 10.1371/journal.pone.0222259

**Published:** 2020-03-25

**Authors:** Gianluigi Giannelli, Armando Santoro, Robin K. Kelley, Ed Gane, Valerie Paradis, Ann Cleverly, Claire Smith, Shawn T. Estrem, Michael Man, Shuaicheng Wang, Michael M. Lahn, Eric Raymond, Karim A. Benhadji, Sandrine Faivre

**Affiliations:** 1 National Institute of Gastroenterology, “s. De Bellis” Research Hospital, Castellana Grotte, Bari, Italy; 2 Humanitas Clinical Institute, Milano, Italy; 3 Helen Diller Family Comprehensive Cancer Center, University of California, San Francisco, California, United States of America; 4 Auckland City Hospital, Auckland, New Zealand; 5 Beaujon Hospital, Clichy, France; 6 Eli Lilly and Company, Windlesham, Surrey, United Kingdom; 7 Eli Lilly and Company, Indianapolis, Indiana, United States of America; 8 BioStat Solutions, Inc., Frederick, Maryland, United States of America; 9 Paris Saint-Joseph Hospital Center, Paris, France; Yonsei University College of Medicine, REPUBLIC OF KOREA

## Abstract

**Background:**

Transforming growth factor beta (TGF-β) signalling is involved in the development of hepatocellular carcinoma (HCC). We followed changes in biomarkers during treatment of patients with HCC with the TGF-βRI/ALK5 inhibitor galunisertib.

**Methods:**

This phase 2 study (NCT01246986) enrolled second-line patients with advanced HCC into one of two cohorts of baseline serum alpha-fetoprotein (AFP): Part A (AFP ≥1.5x ULN) or Part B (AFP <1.5x ULN). Baseline and postbaseline levels of AFP, TGF-β1, E-cadherin, selected miRNAs, and other plasma proteins were monitored.

**Results:**

The study enrolled 149 patients (Part A, 109; Part B, 40). Median OS was 7.3 months in Part A and 16.8 months in Part B. Baseline AFP, TGF-β1, E-cadherin, and an additional 16 plasma proteins (such as M-CSF, IL-6, ErbB3, ANG-2, neuropilin-1, MIP-3 alpha, KIM-1, uPA, IL-8, TIMP-1, ICAM-1, Apo A-1, CA-125, osteopontin, tetranectin, and IGFBP-1) were found to correlate with OS. In addition, a range of miRs were found to be associated with OS. In AFP responders (21% of patients in Part A with decrease of >20% from baseline) versus non-responders, median OS was 21.5 months versus 6.8 months (p = 0.0015). In TGF-β1 responders (51% of all patients) versus non-responders, median OS was 11.2 months versus 5.3 months (p = 0.0036).

**Conclusions:**

Consistent with previous findings, both baseline levels and changes from baseline of circulating AFP and TGF-β1 function as prognostic indicators of survival. Future trials are needed to confirm and extend these results.

## Introduction

Hepatocellular carcinoma (HCC) is the sixth most common cancer worldwide and is increasing in incidence [1]. Systemic treatment options are currently limited to a few agents, such as sorafenib, regorafenib, cabozantinib, or immuno-oncology drugs [2–4]. With an increased understanding of the underlying disease process in HCC, novel treatments are being developed that target specific pathways associated with disease progression [5].

The transforming growth factor beta (TGF-β) signalling pathway was identified as being active in a specific subclass of HCC [6]. However, high circulating levels of TGF-β1 in patients suggest that this pathway may be more broadly active in HCC [7, 8]. In preclinical studies, TGF-β signalling was found to modulate E-cadherin, vimentin, and integrin expression in HCC cells, implying a role in triggering the epithelial-mesenchymal transition (EMT) [9–13]. The small molecule galunisertib, a selective inhibitor of the serine/threonine kinase of the TGF-β receptor type I (TGF-β RI) [14], reversed E-cadherin secretion in highly invasive HCC cell lines and increased the expression of E-cadherin on tumor cells [13]. This change was associated with a reduction in invasion and metastasis [9, 13].

We investigated the activity of galunisertib in two groups of patients with HCC separated by baseline serum alpha fetoprotein (AFP) levels: elevated, or normal to very low. We monitored changes in serum AFP and in plasma TGF-β1 and E-cadherin during treatment. We also assessed other circulating biomarkers, including selected microRNA (miRNA). Where available, we stained tumor tissue samples of patients to determine tumor expression of E-cadherin. All markers were tested for association with overall survival (OS) to determine their potential for prognostic or predictive use in future randomized clinical trials.

## Methods

### Ethics approval

Each institution’s review board approved the study and all patients signed an informed consent document before study participation. The complete list of ethics committee names and reference numbers is in **S2 Table**. The study was conducted according to the principles of good clinical practice, applicable laws and regulations, and the Declaration of Helsinki.

### Patients and study design

This was an open-label, multicentre, two-part phase 2 study in patients aged 18 years or older with histological evidence of HCC (not amenable to curative surgery), Child-Pugh Class A or B7, measurable disease (RECIST 1.1), Eastern Cooperative Oncology Group Performance Status ≤1, and who had received sorafenib and had progressed or were ineligible for sorafenib treatment. Part A included patients with baseline serum AFP≥1.5x upper limit of normal (ULN); these patients were randomly assigned to two cohorts based on initial dose of galunisertib (80 or 150 mg BID) [15]. Part B enrolled patients with AFP<1.5x ULN to receive galunisertib at 150 mg BID. In both parts, galunisertib was given for 14 days followed by 14 days of rest per 28-day cycle. The primary objective was to characterise time-to-tumor progression (TTP) and the effect of treatment on TGF-β-associated plasma biomarkers (TGF-β1, AFP and E-cadherin). The study is registered at ClinicalTrials.gov as NCT01246986.

### Biomarker methods

Plasma or serum samples were analysed for TGF-β1 or AFP levels by ELISA (R&D Systems, DB100B, Minneapolis, Minnesota, USA or Quintiles, Durham, North Carolina, USA, respectively). Except for the initial assessment of serum AFP at the institutional laboratory for the purpose of assignment to Part A or Part B, all baseline and treatment measurements were done at a central laboratory (Quintiles, Durham, North Carolina, USA). The Multianalyte Immunoassay Panel (MAP), using bead array technology developed by Myriad RBM, consisted of approximately 245 plasma proteins; E-cadherin was measured separately (Myriad RBM, Salt Lake City, Utah, USA).

Selected miRNAs in plasma (total 752) were measured using the Exiqon RT microRNA PCR Human panel I+II (Qiagen Inc., Germantown, Maryland, USA). Covance Genomics Laboratories (Seattle, Washington, USA) performed the miR extraction and detection assays.

Tumor tissue, when available, was tested for expression of the following 11 proteins by immunohistochemistry (IHC): cMET, E-cadherin, Glypican-3, PIVKAII, pSMAD2, TGFBR2, AFP, CASPASE3, CD8, CK19 and Ki67 (AAREC Filia Research, Paris, France).

A complete differential blood count was done for lymphocytes, neutrophils and monocytes at a central laboratory (Quintiles, Durham, NC, USA). In a subgroup of patients from Part B, T cell subsets, including CD3+, CD4+, and CD8+, were also evaluated.

### Statistical analyses

AFP, TGF-β1 and E-cadherin levels were measured at baseline and at every 2 weeks after treatment start. When this study was designed, TTP was the recommended endpoint for HCC studies [16]. However, emerging preclinical data indicated that galunisertib treatment reverted EMT [13]. which suggested that evaluable responses and clinical benefit may be delayed, even after treatment discontinuation. Hence, all biomarker responses were assessed using OS and not TTP.

To evaluate if each parameter was prognostic for OS, all patients from both parts were split into two groups, cut at the median of the baseline values of TGF-β1 and E-cadherin and at 400 ng/ml for AFP. Patients were included in the analysis of response if they had at least one evaluable measure at baseline and at any time post-treatment initiation. Patients were considered responders if they achieved >20% decrease from baseline at any visit in the first 6 cycles of treatment, regardless of how many cycles of treatment they completed [17]. For AFP response, only patients in Part A were included in the analysis. For AFP responders, the time of the first occurrence and the subsequent duration of biomarker response was calculated and summarised. For patients who maintained a response at their last assessment, duration was censored at the last visit on which a sample was taken.

OS was summarised descriptively using the Kaplan-Meier method by baseline and subsequent response status of circulating AFP, TGF-β1, and E-cadherin, and used the log-rank test for comparisons.

The MAP developed by Myriad RBM consisted of approximately 245 plasma markers. Potential prognostic markers as measured at baseline were evaluated for their impact on OS. Data were pooled across Parts A and B of the study and patients were split into two groups based on the median value for each biomarker, >median or ≤median. Univariate Cox regression models were used to select markers prognostic for OS with p≤0.001. There was no adjustment for multiplicity given the exploratory nature of the analyses. Spearman’s rank correlation was also estimated between AFP and each of the prognostic markers to verify if any findings may be attributed to an underlying correlation. For identified prognostic markers, changes in the first 6 cycles after treatment with galunisertib were evaluated using mixed effect model repeated measures (MMRM) models. A landmark analysis at 6 cycles was chosen to minimize confounding by multiple measurements in patients on the study longer. Data were log_e_-transformed prior to analysis and the ratio to baseline evaluated, with baseline as a covariate, and fixed effect terms of study part, visit, and the interaction of study part and visit. An AR (1) variance-covariance structure was used to account for repeated measures within a patient.

Lymphocytes, neutrophils and monocytes were evaluated at baseline, weekly during cycle 1, and biweekly during cycle 2 onwards in Parts A and B. T-cell subsets including CD4+, CD8+ and CD3+ were not available for all patients, and were evaluated at baseline for 23/40 patients in Part B. Each laboratory parameter was categorised as low, normal or high at baseline according to central laboratory-defined normal ranges. OS and changes from baseline over the first 6 cycles were summarised and compared using similar methods as for other biomarkers.

For each miRNA, patients were split into four groups by baseline values, using first quartile, median and third quartile as cut-offs. Cox regression was performed using OS as response variable and grouped biomarker as explanatory variable. The statistical significance of the resulting hazard ratios (HRs) were assessed using the likelihood ratio test. The p values were adjusted by Bonferroni correction within each biomarker and then the adjusted p values were again adjusted globally via the false discovery rate method. Median OS and confidence intervals were calculated using the Kaplan-Meier method. For IHC data, similar analyses were performed based on dichotomisation of median for each biomarker.

## Results

### Patients

Patients (n = 149) were enrolled between April 19, 2011 and April 3, 2013, and the cut-off date for the data in this report was May 15, 2015. Of 149 patients who received at least one dose of study drug, 127 (85.2%) were men and 127 (85.2%) were white. Mean age was 64.6 years (range: 31–89 years) and mean weight was 75.4 kg (range: 40.0–120.4 kg) (**S1 Table**) [15]. In Part A (n = 109), 37 received galunisertib at 80 mg BID, and 72 at 150 mg BID; in Part B, 40 received galunisertib at 150 mg BID. A total of 144 patients discontinued study treatment (Part A, 108; Part B, 36). Five patients (Part A,1; Part B, 4) were continuing to receive study treatment at the time of the data cut-off. The most common reasons for treatment discontinuation were progressive disease (98 patients: Part A, 73; Part B, 25), death (20 patients: Part A, 18; Part B, 2) and adverse event (11 patients: Part A, 7; Part B, 4).

### Correlation of biomarkers to survival

The two dose groups of Part A were pooled to enhance the sample size for exploration of biomarker subsets. Median OS was 7.3 months (95% CI: 4.9–10.5) in Part A and 16.8 months (95% CI: 10.5–24.4) in Part B. The log rank p-value for OS for Part A vs Part B was p = 0.001.

**Fig 1:**
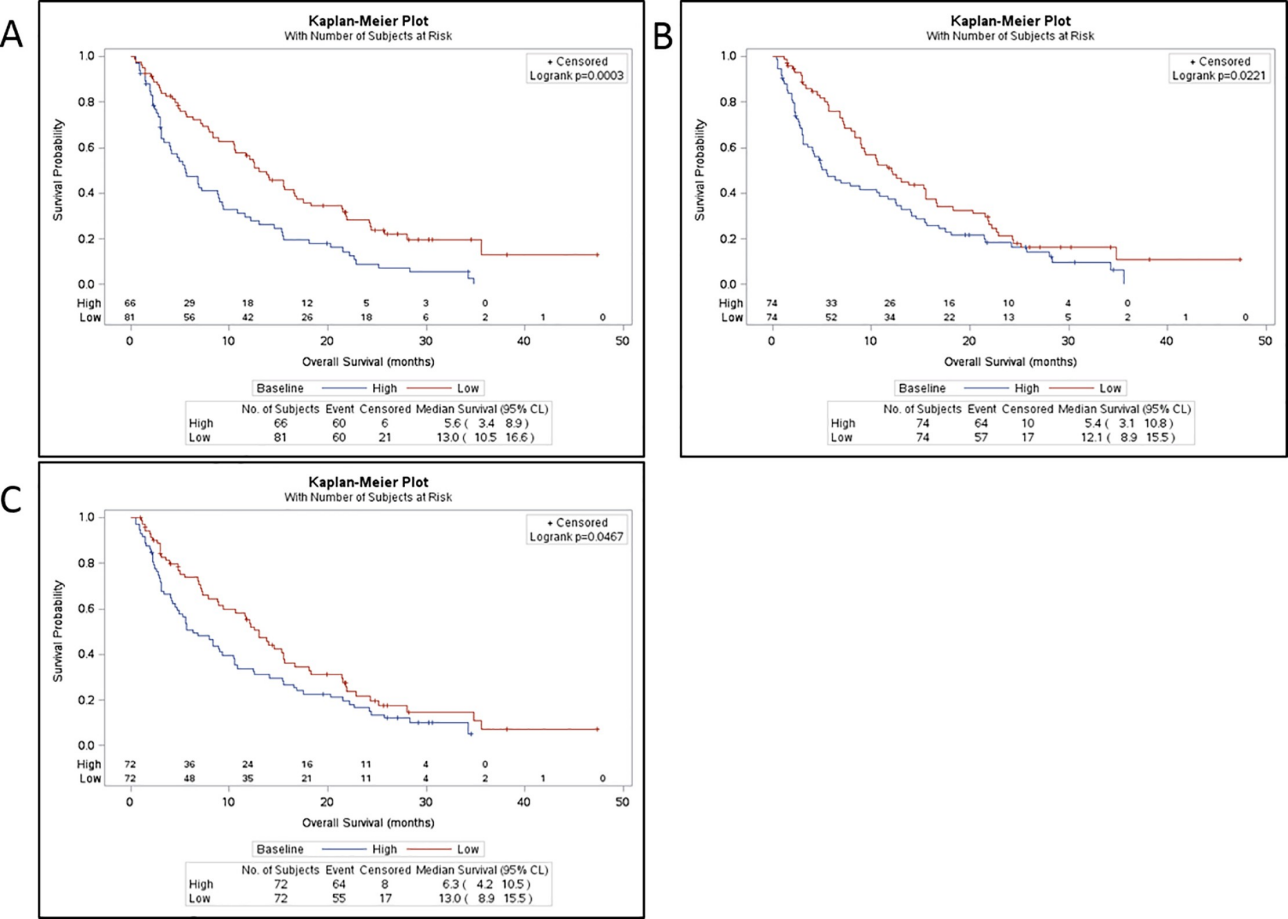
OS by baseline biomarker status (Parts A and B combined). (A) Baseline AFP: low, ≤400 ng/mL, high, >400 ng/mL. (B) Baseline TGF-β1. (C) Baseline E-Cadherin. Baseline levels were split at the median for high versus low comparison for TGF-β1 and E-cadherin.

Combining Parts A and B, and grouping by baseline AFP (>400 ng/mL, ≤400 ng/mL), higher baseline AFP (n = 66) was associated with shorter median OS of 5.6 months (95% CI: 3.4–8.9) compared to 13.0 months (95% CI: 10.5–16.6) for lower baseline AFP (n = 81) (p = 0.0003) (**Table 1, Fig 1A**).

**Table 1:** Evaluation of OS by baseline AFP and TGF-β1 (Parts A and B combined).

Baseline biomarker status	n	Median OS	95% CI	p-value^1^
**Baseline AFP**				
**>400 ng/mL**	66	5.6 mo	3.4–8.9	
**≤400 ng/mL**	81	13.0 mo	10.5–16.6	0.0003
**Baseline TGF-β1^2^**				
**>median**	74	5.4 mo	3.1–10.8	
**≤median**	74	12.1 mo	8.9–15.5	0.022

^1^p-value for difference between baseline biomarker status groups.

^2^Median baseline TGF-β1 was 3.52 ng/mL.

AFP = alpha fetoprotein; mo = months; OS = overall survival; TGF-β1 = transforming growth factor beta 1.

Median baseline TGF-β1 was 3.52 ng/mL. For patients with baseline TGF-β1 above the median (n = 74), median OS was 5.4 months (95% CI: 3.1–10.8) compared to 12.1 months (95% CI: 8.9–15.5) for patients with baseline TGF-β1 ≤median (n = 74), p = 0.022 (**Table 1, Fig 1B**). The results were similar when the data was examined for Part A and Part B separately, where in both parts the median OS was approximately 2-fold longer for the group of patients whose baseline TGF-β1 was ≤median value [15]. This observation is supported by the results from the multivariate Cox regression models combining data across study parts, which provided evidence that TGF-β1 and AFP were independently prognostic for OS (p<0.1) [15].

Median baseline plasma E-cadherin was 6.21 mg/mL. For the group of patients whose baseline E-cadherin was above the median value, median OS was 6.3 months (95% CI: 4.2–10.5) compared to 13.0 months (95% CI: 8.9–15.5) for the group whose baseline E-cadherin ≤median (p = 0.047) (**Fig 1C**).

**Fig 2:**
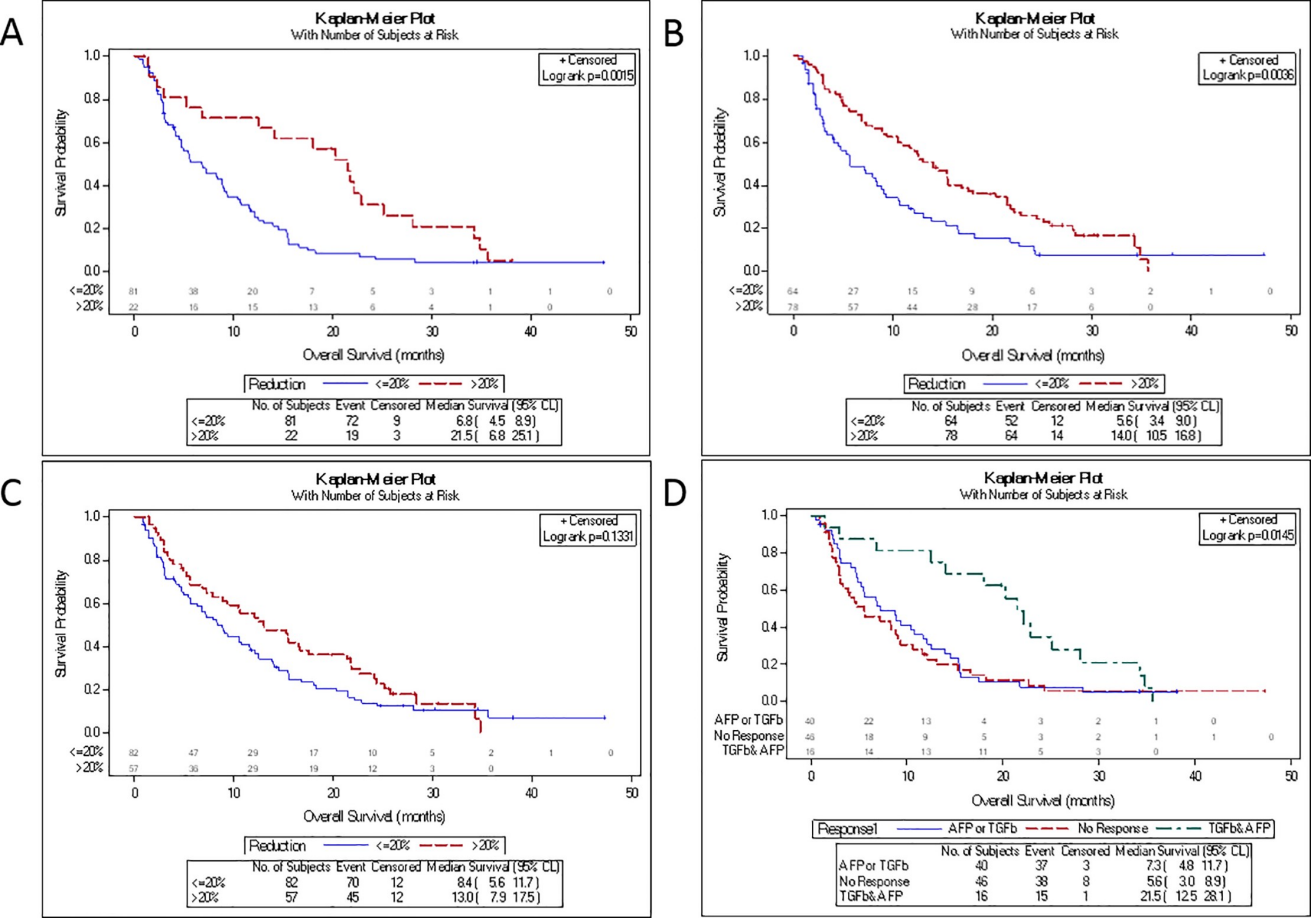
OS by biomarker response status. (A) AFP (Part A only). (B) TGF-β1 (Parts A and B combined). (C) E-cadherin (Parts A and B combined). (D) Overlap of responders for AFP and TGF-β1 (Part A only). Patients were considered a responder if they had >20% decrease from baseline in that biomarker at any time in the first six treatment cycles.

AFP response was evaluated only for patients from Part A. In Part A, 22 (21%) patients demonstrated an AFP response, with a median OS of 21.5 months (95% CI: 6.8–25.1) compared to 6.8 months (95% CI: 4.5–8.9) for patients without an AFP response (p = 0.0015) (**Table 2, Fig 2A**). Median time to first response was 1.4 months, with a median duration of 1 month; however, 5 patients showed a response lasting more than 4 months.

**Table 2:** Evaluation of OS by AFP, TGF-β1 and E-cadherin responses.

	Part A N = 109		Part B N = 40		Total N = 149		
	n (%)	Median OS (mo) (95% CI)	n (%)	Median OS (mo) (95% CI)	n (%)	Median OS (mo) (95% CI)	
**AFP**							
n (patients with data)		103	AFP changes not evaluated for study Part B.				
Responder	22 (21%)	21.5 (6.8, 25.1)					
Non-Responder	81 (79%)	6.8 (4.5, 8.9)					
p-value^a^		0.0015					
Duration of Response^1^		1.0 (0.5-NE)					
Time to 1^st^ response^2^		1.0 (1.0–2.8)					
**TGF-** β**1**							
n (patients with data)		103	39				142
Responder	50 (51%)	11.2 (6.8–14.5)	28 (72%)	21.9 (12.4 –NC)	78 (55%)		14.0 (10.5–16.8)
Non-Responder	53 (49%)	5.3 (3.0–8.9)	11 (28%)	10.5 (1.5–16.5)	64 (45%)		5.6 (3.4, 9.0)
p-value^a^		0.1414		0.0233			0.0036
Duration of Response^1^		0.6 (0.5, 1.4)		0.8 (0.5, 1.0)			0.6 (0.5, 1.0)
Time to 1^st^ response^2^		0.5 (0.5–1.0)		0.5 (0.5–1.4)			0.5 (0.5–1.4)
**E-Cadherin**							
n (patients with data)		100	39				139
Responder	39 (39%)	8.9 (4.9–15.3)	18 (46%)	24.2 (13.0-NE)	57 (41%)		13.0 (7.9–17.5)
Non-Responder	61 (61%)	8.3 (4.5–11.6)	21 (56%)	10.5 (5.6–21.5)	82 (59%)		8.4 (5.6–11.7)
p-value^a^		0.7647		0.0576			0.1331
Duration of Response^1^		1.0 (0.5, 1.0)		0.9 (0.5, 1.0)			1.0 (0.5, 1.0)
Time to 1^st^ response^2^		1.0 (0.5–1.4)		1.0 (0.5–1.4)			1.0 (0.5–1.4)

^a^p-value calculated using the log-rank test for the within study part comparison of the indicated response vs no response comparison

^1^Median (mo) (95% CI)

^2^Median (mo) (25^th^ percentile, 75^th^ percentile)

TGF-β1 response was evaluated for all patients combined. Seventy-eight (55%) patients achieved a TGF-β1 response, with a median OS of 14.0 months (95% CI: 10.5–16.8) compared to 5.6 months (95% CI: 3.4–9.0) for patients without a TGF-β1 response (n = 64) (p = 0.0036) (**Table 2, Fig 2B**). TGF-β1 responses appeared earlier than AFP responses (median time to first response, 0.5 months; median duration, 0.6 months). The shorter duration of response may have been due to the intermittent dosing schedule of galunisertib, with a coinciding fluctuating TGF-β1 response profile (**S1 Fig**).

For patients who had an E-cadherin response, median OS was numerically, but not statistically, longer compared to E-cadherin non-responders (13.0 vs 8.4 mo, p = 0.13) (**Table 2, Fig 2C**).

Of 50 patients in Part A who achieved a TGF-β1 response, 16 achieved an AFP response. The subset of 16 patients achieving both TGF-β1 and AFP responses had median OS of 21.5 months (95% CI: 12.5–28.1) compared to 7.3 months (4.8–11.7) for patients achieving only AFP or TGF-β1 response, and 5.6 months (95% CI: 3.0–8.9) for patients with neither TGF-β1 nor AFP response (**Fig 2D**). These findings suggest that TGF-β1 and AFP may be used as complementary markers for treatment response in future studies.

**Fig 3:**
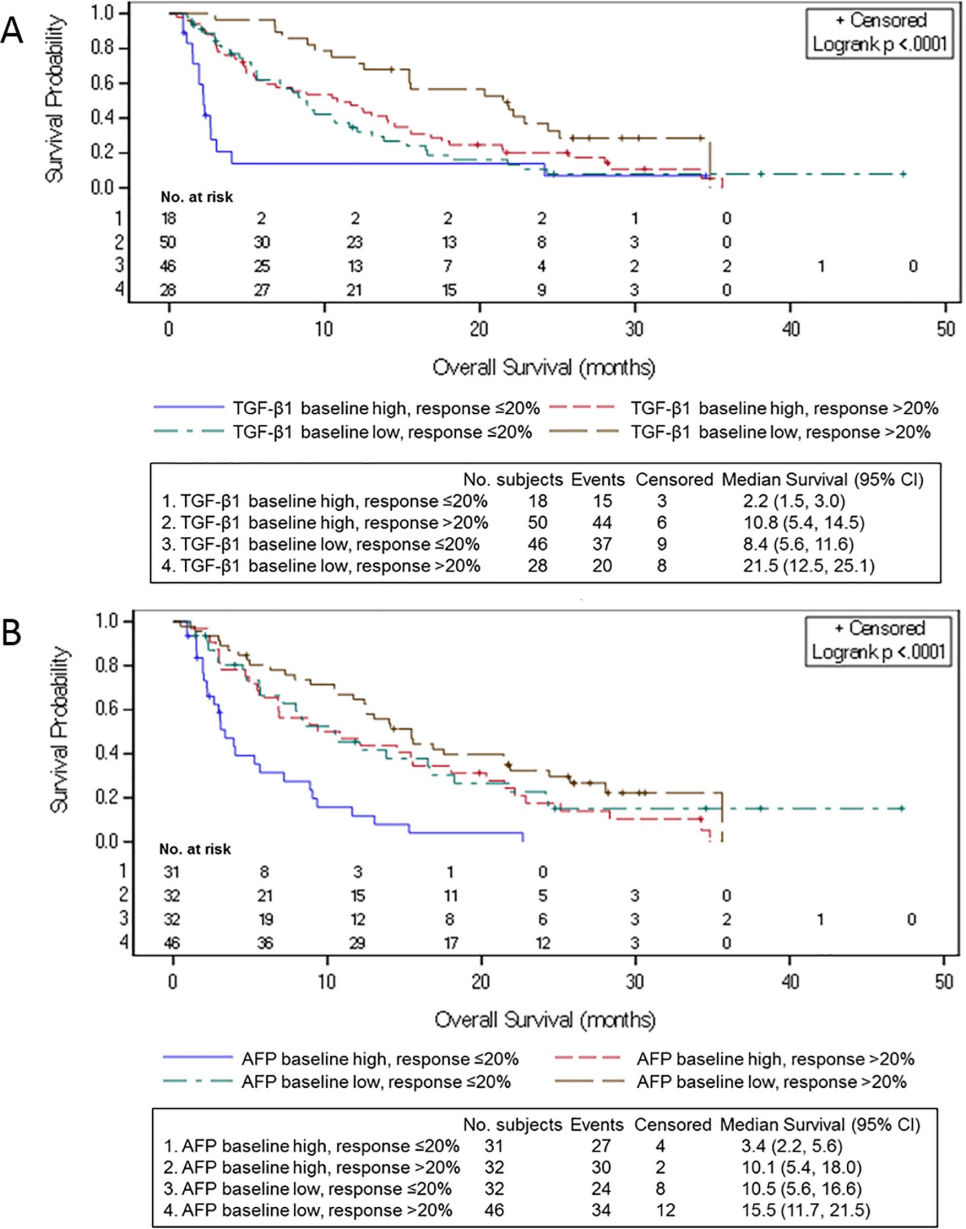
OS by the combination of baseline AFP and TGF-β1, and TGF-β1 response (Parts A and B patients). (A) Baseline TGF-β1 and TGF-β1 response. (B) Baseline AFP and TGF- β1 response.

Finally, we investigated the association between higher TGF-β1 or AFP at baseline, TGF-β1 response, and OS. For patients with higher baseline levels of TGF-β1, 50/68 (73.5%) achieved a TGF-β1 response with median OS of 10.8 months (95% CI: 5.4–14.5) compared to 2.2 months (95% CI: 1.5–3.0) for patients without a TGF-β1 response, p<0.0001 (**Fig 3A**). For patients with higher baseline AFP, 32/63 (50.8%) achieved TGF-β1 response with median OS of 10.1 months (95% CI: 5.4–18.0), compared to 3.4 months (2.2–5.6) for patients without a TGF-β1 response, p<0.0001 (**Fig 3B**).

The number of patients receiving post-discontinuation anticancer therapies was 41 (29%) and the proportion was similar for patients with or without TGF-β1 response. The most common post-discontinuation drug used was sorafenib (10%) followed by oxaliplatin (8%); proportions were similar in Part A and Part B. On average, time on either agent was no longer than 1 cycle. Thus, the relationship of TGF-β1 response to OS was unlikely to be impacted by post-discontinuation therapies.

### Multianalyte immunoassay panel

**Fig 4:**
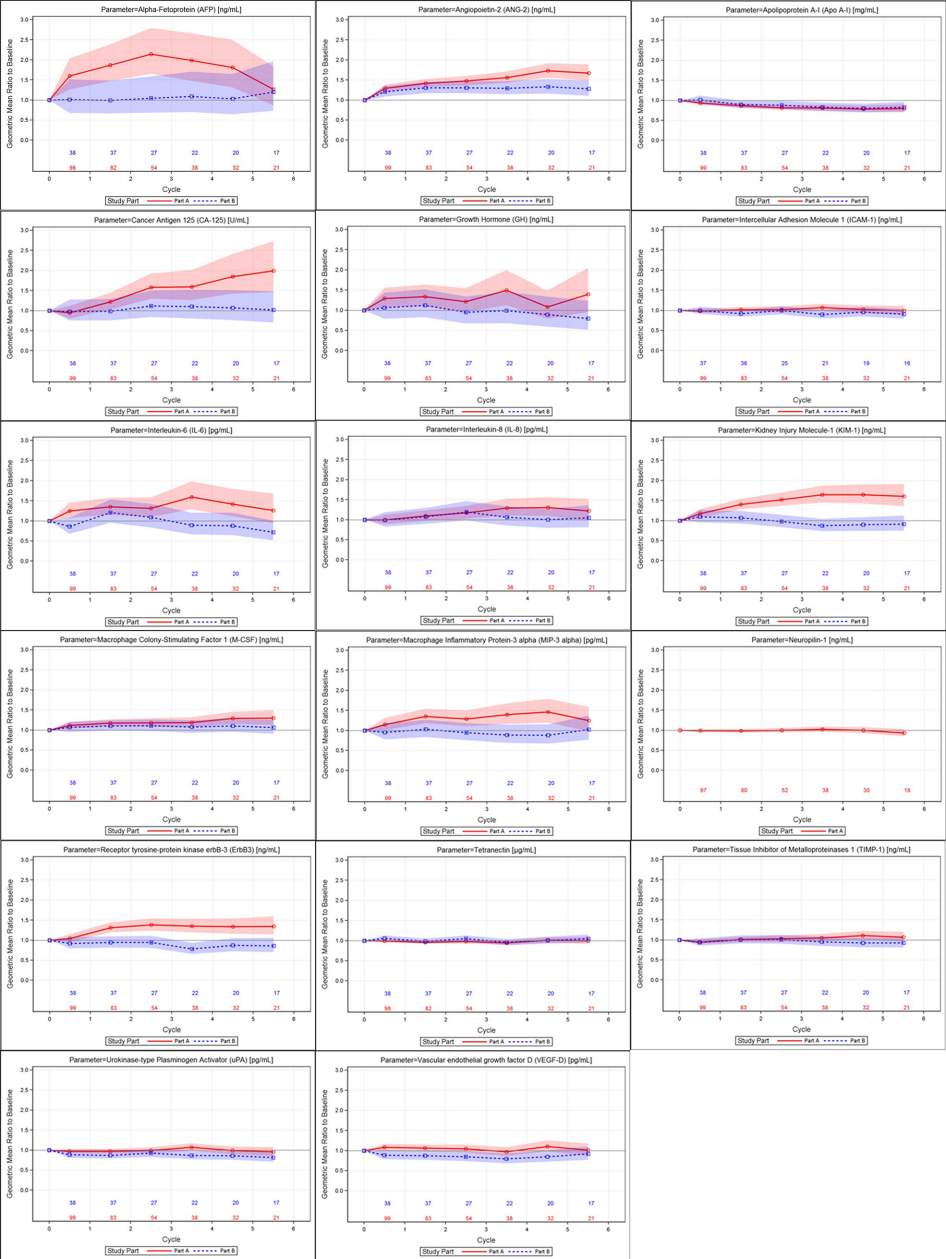
Geometric mean ratio to baseline for selected proteins from the Multianalyte Immunoassay Panel (Parts A and B). Shaded area represents the confidence band and is color-coded by study part.

Of the 245 proteins tested, 33 had >50% of samples below the limit of quantification at baseline and were excluded from analysis, leaving 212 proteins evaluable. Of the 212 proteins, 17 were identified to be prognostic for OS (p<0.001) using the univariate Cox regression model (**Table 3**). Tetranectin and apolipoprotein A-I were found to be associated with longer OS when baseline levels were high (>median). All other parameters were associated with longer OS when baseline levels were low (≤median). Changes in each of the 17 prognostic markers were evaluated after treatment with galunisertib (**Fig 4**), with the following findings:

1. AFP, CA-125 and KIM-1 levels increased by approximately 50% or more after treatment with galunisertib among patients with elevated AFP (≥1.5x ULN) at baseline (Part A), but not in patients with AFP<1.5x ULN at baseline (Part B). Similar effects were also observed but with smaller magnitude for hGH, IL-6, IL-8, MIP-3α and RTK erbB-3.
2. ANGPT2 and M-CSF1 levels showed small increases after treatment for both study parts, with increases more prominent in Part A.
3. APOA1 levels showed modest decreases after treatment in both study parts.
4. VEGF-D showed modest decreases after treatment in Part B, with no trends in Part A.
5. There were no consistent increases or decreases after treatment observed in either study part for ICAM-1, NRP1, tetranectin, TIMP-1 or uPA.
6. Moderate correlation (≥0.5) to AFP was observed for VEGF-D. For all other parameters, correlations were weak.

**Table 3:** Single biomarker analysis: HR for OS by high vs low baseline value and correlation to AFP.

Marker	OS comparison (high vs low)* HR (95% CI)	p-value	Correlation coefficient to AFP
Macrophage Colony-Stimulating Factor 1 (M-CSF)	2.47 (1.71,3.56)	< .0001	0.15
Interleukin-6 (IL-6)	2.37 (1.65,3.42)	< .0001	0.41
Receptor tyrosine-protein kinase erbB-3 (ErbB3)	2.21 (1.54,3.18)	< .0001	0.23
Angiopoietin-2 (ANG-2)	2.16 (1.50,3.10)	< .0001	0.23
Neuropilin-1	2.47 (1.61,3.81)	< .0001	0.04
Alpha Fetoprotein (AFP)	2.10 (1.46,3.03)	< .0001	1.00
Macrophage Inflammatory Protein-3 alpha (MIP-3 alpha)	2.08 (1.45,2.99)	< .0001	0.40
Kidney Injury Molecule-1 (KIM-1)	2.07 (1.44,2.98)	< .0001	0.25
Urokinase-type Plasminogen Activator (uPA)	2.07 (1.43,2.99)	< .0001	0.41
Interleukin-8 (IL-8)	2.03 (1.41,2.91)	< .0001	0.30
Tissue Inhibitor of Metalloproteinases 1 (TIMP-1)	1.93 (1.35,2.77)	0.0003	0.24
Intercellular Adhesion Molecule 1 (ICAM-1)	1.94 (1.35,2.79)	0.0004	0.18
Apolipoprotein A-I (Apo A-I)	0.51 (0.35,0.74)	0.0004	-0.22
Cancer Antigen 125 (CA-125)	1.92 (1.33,2.77)	0.0005	0.04
Osteopontin	1.90 (1.33,2.73)	0.0005	0.18
Tetranectin	0.53 (0.37,0.76)	0.0007	-0.13
Insulin-like Growth Factor-Binding Protein 1 (IGFBP-1)	1.84 (1.28,2.65)	0.0009	0.24

*Marker categorised as high or low at baseline by value above or below the median. Data for Parts A and B combined.

### T-cell subsets and haematological assessments

Baseline numbers of lymphocytes, neutrophils and monocytes were assessed for all patients. Median OS was longer in patients with low to normal (vs high) neutrophils and monocytes (**S2 Fig**). Patients were considered responders if they had a >20% decrease at any time in the first six treatment cycles. Patients who were able to maintain levels of lymphocytes (no decrease >20%) or reduce levels of neutrophils or monocytes (decrease >20%) had improved OS, but differences were not statistically significant (**S3 Fig**). T-cell subsets including CD4+, CD8+ and CD3+ were available for 23 of 40 patients in Part B; no significant associations with OS were identified.

### microRNA

Selected circulating miRNAs (miRs) were measured for 105 patients from Part A at baseline and during treatment at cycle 2, day 14 (C2,D14). The correlation of baseline expression of the measured miRs with OS is shown in **Table 4**. The top eight prognostic miRs were analysed for modulation after treatment, and none were modulated at C2,D14.

**Table 4:** Baseline miR expression associated with OS.

microRNA	p value	Adjusted p value	HR	Size low count	Size high count	FDR
hsa-miR-665	0.00124	0.00372	0.498	53	52	0.129
hsa-miR-320d	0.00125	0.00375	0.440	27	78	0.129
hsa-miR-320a	0.00126	0.00377	0.466	79	26	0.129
hsa-miR-1972	0.00150	0.00451	0.500	53	52	0.129
hsa-miR-451a	0.00161	0.00482	1.99	53	52	0.129
hsa-miR-130b-3p	0.00169	0.00507	0.441	27	78	0.129
hsa-let-7g-5p	0.00170	0.00511	2.25	79	26	0.129
hsa-miR-18a-3p	0.00176	0.00527	1.97	51	50	0.129
hsa-miR-339-5p	0.00420	0.0126	0.485	72	24	0.242
hsa-miR-29b-3p	0.00513	0.0154	1.83	54	51	0.273
hsa-miR-210	0.00707	0.0212	0.494	27	78	0.325
hsa-miR-425-5p	0.00978	0.0293	1.91	79	26	0.378
hsa-miR-346	0.0104	0.0312	0.523	26	76	0.387
hsa-miR-320c	0.0113	0.0339	0.531	27	78	0.400
hsa-miR-877-5p	0.0117	0.0351	0.543	77	28	0.406
hsa-miR-30d-5p	0.0118	0.0353	1.86	79	26	0.407
hsa-miR-363-3p	0.0121	0.0362	1.71	53	52	0.411
hsa-miR-337-3p	0.0145	0.0435	0.546	72	23	0.438
hsa-miR-10b-5p	0.0151	0.0453	1.68	53	52	0.443
hsa-let-7g-5p	0.0152	0.0457	1.89	76	25	0.445

FDR = false discovery rate; HR = hazard ratio; size low count = number of patients with values below the cut-off; size high count = no. of patients with values above the cut-off.

### Tumor tissue IHC

No analysis was performed for proteins AFP, CK19, CASPASE3, Ki67 and CD8 because of insufficient samples. For AFP, only 10% of samples scored for any detectable signal. cMET, E-cadherin, Glypican-3, PIVKAII, pSMAD2 and TGFBR2 data were analysed, but patients with evaluable samples were limited (Part A: 30–44 samples and Part B: 12–22 samples). Tumor tissue expression of proteins AFP, TGFBR2, pSMAD2, E-cadherin, PIVKAII, cMET and Glypican-3 was analysed to determine relationship to clinical outcomes in Part A or Part B, separately or combined. Higher expression of E-cadherin (>median IHC score of 85) was associated with shorter OS in Part B (n = 20, HR = 3.83, unadjusted p = 0.029), but not in Parts A and B combined (n = 61, unadjusted p = 0.412). However, when using a higher cut-off (75% cut point IHC score of 100), higher expression of E-cadherin was associated with a shorter OS (n = 61, HR = 2.60, unadjusted p = 0.006) in Parts A and B combined. The other tissue markers did not show an association with survival.

## Discussion

In the present study, we observed that median OS was 7.3 months for patients in Part A and 16.8 months for patients in Part B. These survival times were similar to previously reported results for second-line patients with HCC treated with novel agents [18]. However, in contrast to previous studies, such as the SHARP trial where no biomarker was established to help with the dosing of sorafenib in HCC patients, we used a predefined inclusion of patients based on their serum levels of AFP [19], which is a prognostic covariate in scoring systems such as CLIP [20]. High baseline levels of plasma TGF-β1 and E-cadherin and low tumor expression of E-cadherin have also been associated with poor outcome [13, 21].

First, we showed that higher baseline serum AFP and plasma TGF-β1 levels were indeed associated with shorter OS as previously reported [8, 22]. We also observed that higher plasma E-cadherin levels at baseline were associated with shorter OS (**Fig 1**). Next, we showed that patients who had a response (decrease from baseline >20%) in both AFP and TGF-β1 during galunisertib therapy had a significantly prolonged OS (median OS 21.5 months). Responses in plasma TGF-β1 or E-cadherin alone were less significant, with E-cadherin showing the least effect (**Fig 2**). Although the downstream effect of TGF-β1 on E-cadherin production and expression is well reported [23, 24], there are no studies reporting the molecular timing regulating such interactions. Even less investigated is the relationship between TGF-β1 pathway inhibition and downstream effect on E-cadherin.

There was a greater proportion of TGF-β1 responders among patients with higher baseline plasma TGF-β1 (73.5%; 50/68) compared to those with lower baseline plasma TGF-β1 (37.8%; 28/74). In contrast, TGF-β1 response was similar in the two baseline AFP cohorts: 50.8% (32/63) of patients in Part A with higher AFP levels and 59.0% (46/78) of patients in Part B with lower AFP levels had a TGF-β1 response. Survival was longest in patients with low baseline TGF-β1 or AFP who experienced a TGF-β1 response during treatment (**Fig 3**).

Since plasma TGF-β1 levels may serve as a biomarker to assess clinical activity of galunisertib in future randomized trials in a larger HCC population, we assessed the rapidity of the onset of TGF-β1 response (**S1 Fig, Table 2**). Responses occurred during the first cycle, with TGF-β1 response generally occurring first, followed by E-cadherin and AFP responses. The duration of biomarker response was longest for AFP, perhaps making this marker more reliable for when measurements are infrequent. However, plasma TGF-β1 responses may look different if the TGF-β inhibitor were to be given continuously and not intermittently as done for galunisertib.

The fact that TGF-β1 levels were elevated in a large number of patients suggests that inflammation may have impaired the ability of immune cells to respond to tumor antigens. This was supported by the data from the larger protein panel (**Fig 4, Table 3**). IL-6 and IL-8 correlated with TGF-β1, as did factors associated with liver function and vascular responses.

Of the eight circulating miRs associated with OS in this study, miR-320a, miR-130b and miR-18a have previously been detected in plasma or serum of HCC patients [25–27], and miR-320a, miR-451a, miR-130b and miR-let-7g were previously detected in normal liver and HCC tumor samples (TCGA data: https://cancergenome.nih.gov/). Thus, several of the top circulating miRs associated with OS may originate from liver or the hepatic tumor. Two miRs, miR-451a and miR-130b, were previously shown to be prognostic markers in HCC [28, 29]; we also found an association with OS (**Table 4**).

Finally, tumor tissue analyses were available from a subset of patients. We expected that patients with high plasma E-cadherin levels would also have low tissue expression of E-cadherin and shorter survival [30]. However, we observed that patients with high tumor E-cadherin expression had shorter survival. This assessment was limited by two major facts: (1) the samples originated from archival specimen and were not taken prior to treatment, and (2) the evaluable sample size was too small to determine the variability and to make correlations to the plasma levels of E-cadherin.

A possible limitation of our study is the way in which patients were assigned as having “low” versus “high” levels of the biomarkers TGF-β1 and E-cadherin at baseline, by using the median of the study population. It would be more ideal to determine “low” versus “high” in representative groups of healthy volunteers, other disease states, or other cancers.

In conclusion, in a subset of patients with advanced HCC treated with the novel TGF-βRI/ALK5 inhibitor galunisertib, decreases in circulating AFP and TGF-β1 levels were associated with longer OS. Future randomized studies will examine the effect of treatment with galunisertib or other TGF-β1 inhibitors in patients with poor prognosis as defined by high baseline levels of AFP or plasma TGF-β1.

## Supporting information

S1 Table Demographic and baseline characteristics. (DOCX)

S2 Table List of ethical review boards. (DOCX)

S1 Fig Patient profile plots of percentage change from baseline in the first 12 treatment cycles, and box plots of percentage change from baseline in the first 2 cycles. All plots are color-coded by whether the patient achieved TGF-β1 response (decrease from baseline >20% at any time in the first 6 cycles). Y-axis truncated for visual purposes. (A) and (B) Serum AFP (Part A only). (C) and (D) Plasma TGF-β1 (Part A and B combined). (E) and (F) Plasma E-cadherin (Parts A and B combined). Box plots: symbol = mean, bar = median, box = interquartile range, error bars = high and low values.
(TIF)

S2 Fig Overall survival by baseline laboratory defined normal ranges (Parts A and B combined). (A) Lymphocytes. (B) Neutrophils. (C) Monocytes.
(TIF)

S3 Fig Overall survival by response in cell number (decrease from baseline >20% in the first 6 cycles of treatment, Parts A and B combined). (A) Lymphocytes. (B) Neutrophils. (C) Monocytes.
(TIF)

## References

[1] Mittal S, El-Serag HB: Epidemiology of hepatocellular carcinoma: consider the population. J Clin Gastroenterol 2013; 47 Suppl:S2–6. 23632345 10.1097/MCG.0b013e3182872f29 PMC3683119

[2] Bruix J, Qin S, Merle P, Granito A, Huang YH, Bodoky G et al: Regorafenib for patients with hepatocellular carcinoma who progressed on sorafenib treatment (RESORCE): a randomised, double-blind, placebo-controlled, phase 3 trial. Lancet 2017; 389:56–66. 10.1016/S0140-6736(16)32453-9 27932229

[3] Kudo M: Molecular Targeted Agents for Hepatocellular Carcinoma: Current Status and Future Perspectives. Liver Cancer 2017; 6:101–112. 10.1159/000452138 28275577 PMC5340214

[4] Woo HY, Yoo SY, Heo J: New chemical treatment options in second-line hepatocellular carcinoma: what to do when sorafenib fails? Expert Opin Pharmacother 2017; 18:35–44. 10.1080/14656566.2016.1261825 27849399

[5] Shao YY, Hsu CH, Cheng AL: Predictive biomarkers of antiangiogenic therapy for advanced hepatocellular carcinoma: where are we? Liver Cancer 2013; 2:93–107. 10.1159/000343845 24159601 PMC3740718

[6] Hoshida Y, Nijman SM, Kobayashi M, Chan JA, Brunet JP, Chiang DY et al: Integrative transcriptome analysis reveals common molecular subclasses of human hepatocellular carcinoma. Cancer Res 2009; 69:7385–7392. 10.1158/0008-5472.CAN-09-1089 19723656 PMC3549578

[7] Giannelli G, Fransvea E, Marinosci F, Bergamini C, Colucci S, Schiraldi O et al: Transforming growth factor-beta1 triggers hepatocellular carcinoma invasiveness via alpha3beta1 integrin. Am J Pathol 2002; 161:183–193. 10.1016/s0002-9440(10)64170-3 12107103 PMC1850694

[8] Lin TH, Shao YY, Chan SY, Huang CY, Hsu CH, Cheng AL: High Serum Transforming Growth Factor-beta1 Levels Predict Outcome in Hepatocellular Carcinoma Patients Treated with Sorafenib. Clinical cancer research: an official journal of the American Association for Cancer Research 2015; 21:3678–3684. 10.1158/1078-0432.CCR-14-1954 25977342

[9] Giannelli G, Bergamini C, Fransvea E, Sgarra C, Antonaci S: Laminin-5 with transforming growth factor-beta1 induces epithelial to mesenchymal transition in hepatocellular carcinoma. Gastroenterology 2005; 129:1375–1383. 10.1053/j.gastro.2005.09.055 16285938

[10] Serova M, Garbay D, Riveiro ME, Bieche I, Bouattour M, Raymond E et al: Targeting of TGF-beta signaling results in decreased motility and invasion in parental and mulitkinase inhibitor-insensitive hepatocacinoma cells. In: ILCA Annual Conference 2011: 2011; Brussels: International Liver Cancer Association (ILCA); 2011.

[11] Fransvea E, Angelotti U, Antonaci S, Giannelli G: Blocking transforming growth factor-beta up-regulates E-cadherin and reduces migration and invasion of hepatocellular carcinoma cells. Hepatology 2008; 47:1557–1566. 10.1002/hep.22201 18318443

[12] Fransvea E, Mazzocca A, Antonaci S, Giannelli G: Targeting transforming growth factor (TGF)-betaRI inhibits activation of beta1 integrin and blocks vascular invasion in hepatocellular carcinoma. Hepatology 2009; 49:839–850. 10.1002/hep.22731 19115199

[13] Fransvea E, Mazzocca A, Santamato A, Azzariti A, Antonaci S, Giannelli G: Kinase activation profile associated with TGF-beta-dependent migration of HCC cells: a preclinical study. Cancer chemotherapy and pharmacology 2011; 68:79–86. 10.1007/s00280-010-1459-x 20844878

[14] Herbertz S, Sawyer JS, Stauber AJ, Gueorguieva I, Driscoll KE, Estrem ST et al: Clinical development of galunisertib (LY2157299 monohydrate), a small molecule inhibitor of transforming growth factor-beta signaling pathway. Drug design, development and therapy 2015; 9:4479–4499. 10.2147/DDDT.S86621 26309397 PMC4539082

[15] Faivre S, Santoro A, Kelley RK, Gane E, Costentin CE, Gueorguieva I et al: Novel Transforming Growth Factor Beta Receptor I Kinase Inhibitor Galunisertib (LY2157299) in Advanced Hepatocellular Carcinoma. Liver Int 2019 8;39(8):1468–1477. 10.1111/liv.14113 30963691

[16] Llovet JM, Di Bisceglie AM, Bruix J, Kramer BS, Lencioni R, Zhu AX et al: Design and endpoints of clinical trials in hepatocellular carcinoma. J Natl Cancer Inst 2008; 100:698–711. 10.1093/jnci/djn134 18477802

[17] Personeni N, Bozzarelli S, Pressiani T, Rimassa L, Tronconi MC, Sclafani F et al: Usefulness of alpha-fetoprotein response in patients treated with sorafenib for advanced hepatocellular carcinoma. J Hepatol 2012; 57:101–107. 10.1016/j.jhep.2012.02.016 22414760

[18] Llovet JM, Hernandez-Gea V: Hepatocellular carcinoma: reasons for phase III failure and novel perspectives on trial design. Clinical cancer research: an official journal of the American Association for Cancer Research 2014; 20:2072–2079. 10.1158/1078-0432.CCR-13-0547 24589894

[19] Llovet JM, Ricci S, Mazzaferro V, Hilgard P, Gane E, Blanc JF et al: Sorafenib in advanced hepatocellular carcinoma. N Engl J Med 2008; 359:378–390. 10.1056/NEJMoa0708857 18650514

[20] Kudo M, Chung H, Osaki Y: Prognostic staging system for hepatocellular carcinoma (CLIP score): its value and limitations, and a proposal for a new staging system, the Japan Integrated Staging Score (JIS score). J Gastroenterol 2003; 38:207–215. 10.1007/s005350300038 12673442

[21] Giannelli G, Santoro A, Kelley RK, Merle P, Gane E, Douillard J-Y et al: Phase 2 Study of the Oral Transforming Growth Factor-Beta (TGF-beta) Receptor I Kinase Inhibitor LY2157299. In: 7th ILCA (International Liver Cancer Association) Annual Conference: 2013; Washington, D.C., USA; 2013.

[22] Raoul JL, Bruix J, Greten TF, Sherman M, Mazzaferro V, Hilgard P et al: Relationship between baseline hepatic status and outcome, and effect of sorafenib on liver function: SHARP trial subanalyses. J Hepatol 2012; 56:1080–1088. 10.1016/j.jhep.2011.12.009 22245896

[23] Vogelmann R, Nguyen-Tat MD, Giehl K, Adler G, Wedlich D, Menke A: TGFbeta-induced downregulation of E-cadherin-based cell-cell adhesion depends on PI3-kinase and PTEN. J Cell Sci 2005; 118:4901–4912. 10.1242/jcs.02594 16219695

[24] Xu J, Lamouille S, Derynck R: TGF-beta-induced epithelial to mesenchymal transition. Cell Res 2009; 19:156–172. 10.1038/cr.2009.5 19153598 PMC4720263

[25] Liu AM, Yao TJ, Wang W, Wong KF, Lee NP, Fan ST et al: Circulating miR-15b and miR-130b in serum as potential markers for detecting hepatocellular carcinoma: a retrospective cohort study. BMJ Open 2012; 2:e000825 10.1136/bmjopen-2012-000825 22403344 PMC3308260

[26] Sohn W, Kim J, Kang SH, Yang SR, Cho JY, Cho HC et al: Serum exosomal microRNAs as novel biomarkers for hepatocellular carcinoma. Exp Mol Med 2015; 47:e184 10.1038/emm.2015.68 26380927 PMC4650928

[27] Wen Y, Han J, Chen J, Dong J, Xia Y, Liu J et al: Plasma miRNAs as early biomarkers for detecting hepatocellular carcinoma. Int J Cancer 2015; 137:1679–1690. 10.1002/ijc.29544 25845839

[28] Huang JY, Zhang K, Chen DQ, Chen J, Feng B, Song H et al: MicroRNA-451: epithelial-mesenchymal transition inhibitor and prognostic biomarker of hepatocelluar carcinoma. Oncotarget 2015; 6:18613–18630. 10.18632/oncotarget.4317 26164082 PMC4621914

[29] Wang WY, Zhang HF, Wang L, Ma YP, Gao F, Zhang SJ et al: High expression of microRNA-130b correlates with poor prognosis of patients with hepatocellular carcinoma. Diagn Pathol 2014; 9:160 10.1186/s13000-014-0160-5 25123453 PMC4141946

[30] Chen J, Zhao J, Ma R, Lin H, Liang X, Cai X: Prognostic significance of E-cadherin expression in hepatocellular carcinoma: a meta-analysis. PLoS One 2014; 9:e103952 10.1371/journal.pone.0103952 25093414 PMC4122395

