# Correction: Biomarkers and overall survival in patients with advanced hepatocellular carcinoma treated with TGF-βRI inhibitor galunisertib

**DOI:** 10.1371/journal.pone.0235580

**Published:** 2020-06-25

**Authors:** The PLOS ONE Staff

The Data Availability statement for this paper is incorrect. The publisher apologizes for the error. The correct statement is: Data are available upon request at vivli.org. Access to the data is provided after a proposal has been approved by an independent review committee identified for this purpose and after receipt of a signed data sharing agreement. Data and documents, including the study protocol, statistical analysis plan, clinical study report, blank or annotated case report forms, will be provided in a secure data sharing environment. For details on submitting a request, see the instructions provided at www.vivli.org. In your request, please include the following accession/reference numbers so that we can identify the dataset you're trying to access: NCT01246986.
