# Correction: Biomarkers and overall survival in patients with advanced hepatocellular carcinoma treated with TGF-βRI inhibitor galunisertib

**DOI:** 10.1371/journal.pone.0253671

**Published:** 2021-06-17

**Authors:** Gianluigi Giannelli, Armando Santoro, Robin K. Kelley, Ed Gane, Valerie Paradis, Ann Cleverly, Claire Smith, Shawn T. Estrem, Michael Man, Shuaicheng Wang, Michael M. Lahn, Eric Raymond, Karim A. Benhadji, Sandrine Faivre

There is an error in affiliation 2 for the second author Armando Santoro. The correct affiliation 2 is: Humanitas Clinical and Research Center–IRCCS, Humanitas Cancer Center, Milan, Italy. An additional affiliation is also missing for the second author. Armando Santoro is also affiliated with Humanitas University, Department of Biomedical Sciences, Milan, Italy.
