# Supplementary material for: Biomarkers and overall survival in patients with advanced hepatocellular carcinoma treated with TGF-βRI inhibitor galunisertib
Source: PLoS One. 2020 Mar 25;15(3):e0222259. doi: 10.1371/journal.pone.0222259 (PMC7094874; doi:10.1371/journal.pone.0222259)
Supplement: S1 Table — (DOCX) [file pone.0222259.s001.docx]

**S1 Table. Demographic and Baseline Characteristics**

| **Parameter** | **n=149** |
| --- | --- |
|  |  |
| Male, n | 127 |
| Age, median (range), yr | 65 (31–89) |
| Ethnicity, n | |
| White (non-Asian) | 127 |
| Asian | 9 |
| Other/Missing | 7 |
| Prior sorafenib, n | |
| Yes/No | 123/26 |
| Eastern Cooperative Oncology Group Performance Status, n | |
| 0/1 | 84/65 |
| Child Pugh score, n | |
| 5/6/7 | 67/61/21 |
| Pre-existing liver disease, n | |
| Hepatitis C | 36 |
| Hepatitis B | 30 |
| Alcohol use | 30 |
| Steatosis (non-alcoholic fatty liver disease) | 13 |
| Haemochromatosis | 6 |
| Other (no cause provided) | 20 |
| Multiple | 14 |
| Liver transplant, n | 4 |
| Portal vein thrombosis, n |  |
| Yes/No/Missing | 39/109/1 |
| Tumor morphology, n | |
| Unimodular | 19 |
| Mulitmodular | 100 |
| Massive | 28 |
| Missing | 2 |
| Baseline alpha fetoprotein | |
| >400 ng/mL | 66 |
| Missing | 1 |
