# Supplementary material for: Biomarkers and overall survival in patients with advanced hepatocellular carcinoma treated with TGF-βRI inhibitor galunisertib
Source: PLoS One. 2020 Mar 25;15(3):e0222259. doi: 10.1371/journal.pone.0222259 (PMC7094874; doi:10.1371/journal.pone.0222259)
Supplement: S2 Table — (DOCX) [file pone.0222259.s002.docx]

# S2 Table. List of ethics review committees

| Country Code | Ethics Review Board Name | City/Town | State/Country |
| --- | --- | --- | --- |
| CH | Commission cantonale d'éthique de la recherche sur l'être hu | Lausanne | Switzerland |
| DE | Ethikkommission bei der Landesärztekammer Rheinland-Pfalz | Mainz | Germany |
| ES | Hospital Universitari Vall d'Hebron | Barcelona | Spain |
| FR | CPP Ile de France VIII Hôpital Ambroise Paré | Boulogne-Billancourt | France |
| IT | Comitato Etico dell'Azienda Osp.Policlinico Consorziale Bari | Bari | Italy |
| IT | COMITATO ETICO INDIPENDENTE ISTITUTO CLINICO HUMANITAS | Rozzano | Italy |
| IT | Istituto Scientifico Romagnolo - Studio e la Cura dei Tumori | Meldola | Italy |
| IT | Comitato Etico Fondazione Policlinico Univ. A.Gemelli | Roma | Italy |
| IT | Comitato Etico del Policlinico Vittorio Emanuele (Catania 1) | Catania | Italy |
| NZ | Health and Disability Ethics Committee, Northern Y Regional Ethics Committee, Ministry of Health | Hamilton | New Zealand |
| US | Lahey Clinic Medical Center | Burlington | MA/USA |
| US | Indiana University Health | Indianapolis | IN/USA |
| US | University of California, San Francisco | San Francisco | CA/USA |
| US | Northwestern University | Chicago | IL/USA |
| US | Western Institutional Review Board - WIRB | Olympia | WA/USA |
| US | MD Anderson Cancer Center Orlando | Orlando | FL/USA |
| US | Weill Cornell Medical College | New York | NY/USA |
| US | Western Institutional Review Board - WIRB | Olympia | WA/USA |
| US | Western Institutional Review Board - WIRB | Puyallup | WA/USA |
| US | Georgetown University Medical Center | Washington | DC/USA |
| US | Thomas Jefferson University | Philadelphia | PA/USA |
| US | Memorial Sloan Kettering Cancer Center | New York | NY/USA |
