# Supplementary figures and images for: Biomarkers and overall survival in patients with advanced hepatocellular carcinoma treated with TGF-βRI inhibitor galunisertib
Source: PLoS One. 2020 Mar 25;15(3):e0222259. doi: 10.1371/journal.pone.0222259 (PMC7094874; doi:10.1371/journal.pone.0222259)

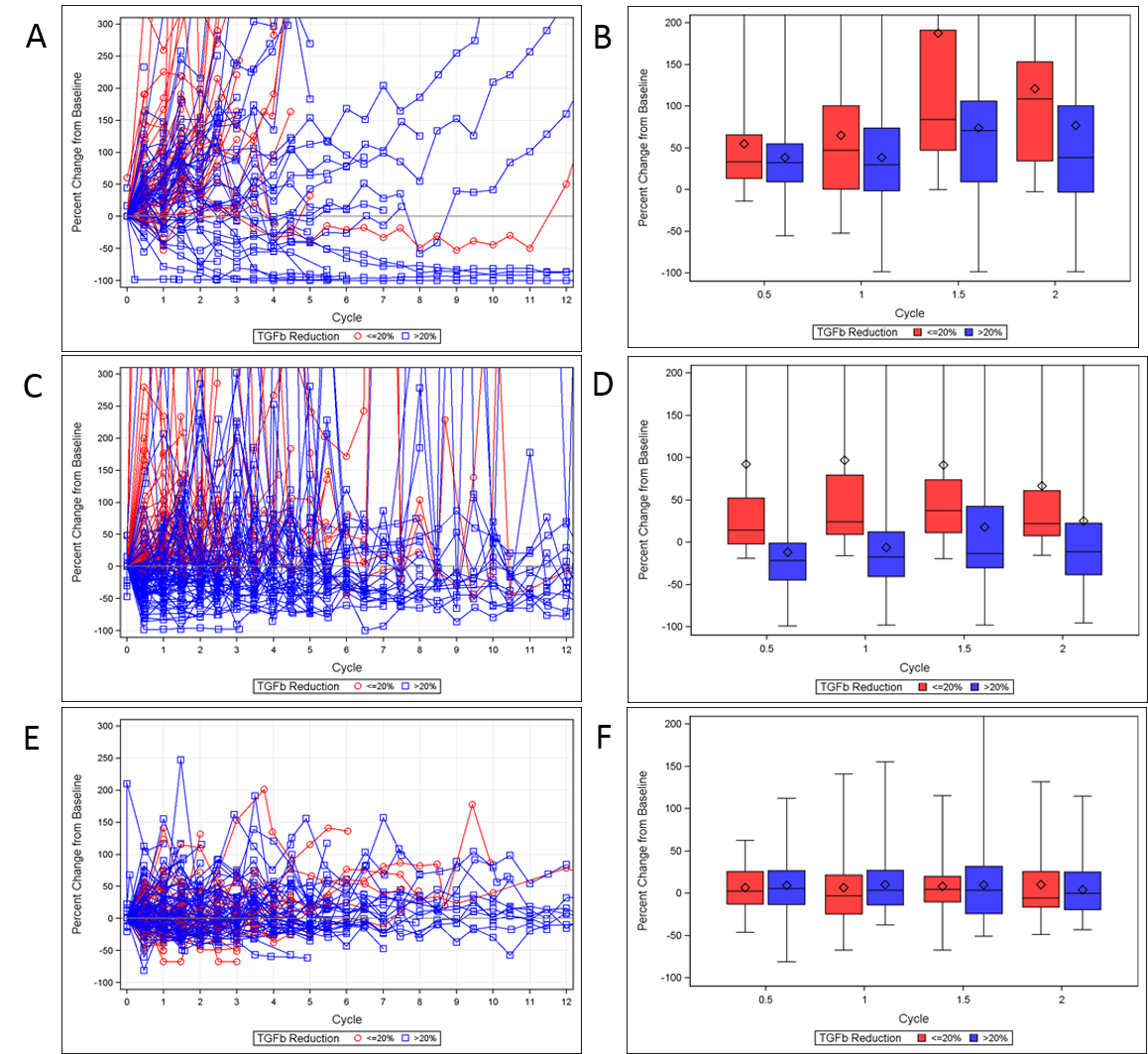

Supplement: S1 Fig — All plots are color-coded by whether the patient achieved TGF-β1 response (decrease from baseline >20% at any time in the first 6 cycles). Y-axis truncated for visual purposes. (A) and (B) Serum AFP (Part A only). (C) and (D) Plasma TGF-β1 (Part A and B combined). (E) and (F) Plasma E-cadherin (Parts A and B combined). Box plots: symbol = mean, bar = median, box = interquartile range, error bars = high and low values. (TIF) [file pone.0222259.s003.tif]

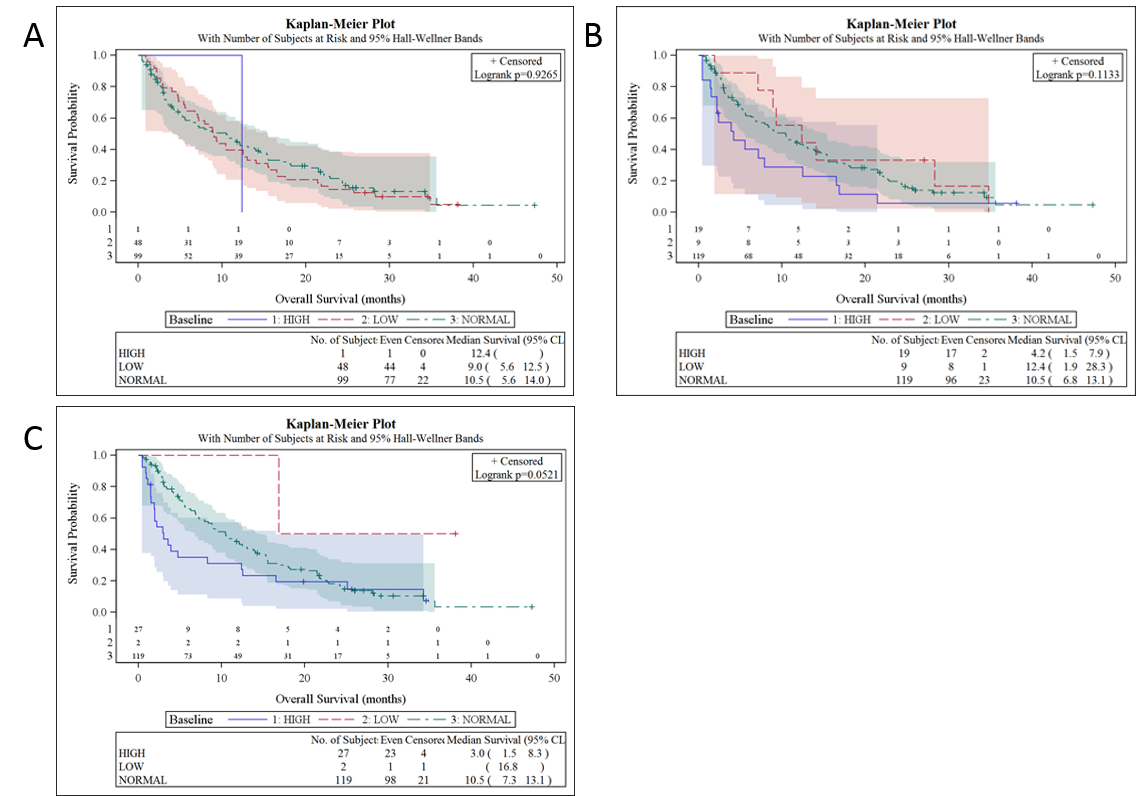

Supplement: S2 Fig — Overall survival by baseline laboratory defined normal ranges (Parts A and B combined). (A) Lymphocytes. (B) Neutrophils. (C) Monocytes. (TIF) [file pone.0222259.s004.tif]

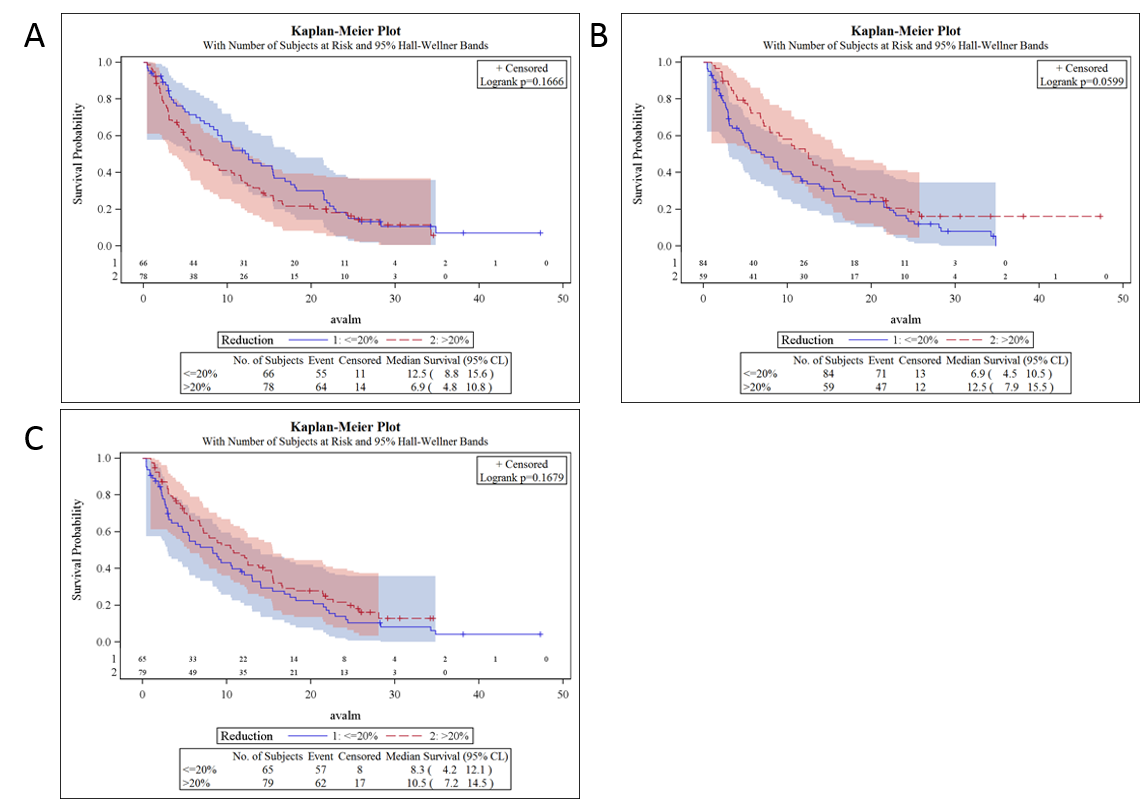

Supplement: S3 Fig — Overall survival by response in cell number (decrease from baseline >20% in the first 6 cycles of treatment, Parts A and B combined). (A) Lymphocytes. (B) Neutrophils. (C) Monocytes. (TIF) [file pone.0222259.s005.tif]
